# Epigenome-wide association study of plasma homocysteine in a Korean cohort highlights imprinted genomic domains as top regional methylation signals

**DOI:** 10.1186/s13148-026-02227-5

**Published:** 2026-08-12

**Authors:** Jaeeun Yoo

**Affiliations:** Department of Laboratory Medicine, Incheon St. Mary’s Hospital, The Catholic University of Korea, 56 Dongsu-ro, Bupyeong-gu, Incheon, 21431 Republic of Korea

**Keywords:** Epigenome-wide association study, Homocysteine, One-carbon metabolism, L3MBTL1, PEG3, BLCAP, NNAT, Imprinting, Korean cohort, KoGES, DNA methylation

## Abstract

**Background:**

Elevated plasma homocysteine impairs DNA methylation capacity by raising the intracellular S-adenosylhomocysteine (SAH)/S-adenosylmethionine (SAM) ratio, thereby inhibiting DNA methyltransferases. Prior epigenome-wide association studies (EWAS) of homocysteine have been conducted almost exclusively in European populations, and data from East Asian populations remain limited.

**Methods:**

We performed an EWAS of fasting plasma homocysteine in 1,510 Korean adults from the Korea Genome and Epidemiology Study (KoGES/KARE) cohort using the Illumina MethylationEPIC 850 K array. Blood cell type proportions were estimated by reference-based deconvolution (EpiDISH, 12 cell types) and included as covariates alongside age, sex, BMI, smoking status, array position, and global mean methylation. We tested 845,023 CpG sites for association with log-transformed homocysteine using ordinary least squares regression. Differentially methylated regions (DMRs) were identified by positional clustering and Stouffer combined Z-scores with Benjamini-Hochberg FDR correction. We additionally performed sensitivity analyses adjusting for alcohol consumption, serum creatinine, folate, vitamin B12, and *MTHFR* C677T genotype.

**Results:**

The study population had a mean homocysteine of 14.19 ± 5.51 µmol/L and a hyperhomocysteinemia prevalence of 22.7% (> 16 µmol/L). The genomic inflation factor was λ = 1.227. Three probes on chromosome 1 reached FDR < 0.05. Regional analysis identified three known imprinted loci among the top-ranked DMRs: the *L3MBTL1* locus (chr20q12, 31 CpGs, Stouffer *p* = 2.0 × 10^−20^), the *PEG3*/*ZIM2*/*MIMT1* domain (chr19q13.43, 40 CpGs, *p* = 5.2 × 10^−20^), and the *BLCAP*/*NNAT* locus (chr20q11.21, 55 CpGs, *p* = 1.4 × 10^−15^). The *L3MBTL1* and *PEG3* domains remained significant after adjustment for folate, vitamin B12, *MTHFR* C677T, alcohol, and creatinine (e.g., *PEG3* FDR = 5.1 × 10^−15^ after *MTHFR* adjustment); the *BLCAP*/*NNAT* signal was attenuated by folate/B12 adjustment. The three chromosome-1 probes lost epigenome-wide significance after *MTHFR* C677T adjustment (FDR ≈ 0.4), consistent with *cis*-meQTL confounding.

**Conclusions:**

This EWAS of plasma homocysteine in a Korean cohort—one of the largest population-based homocysteine EWAS reported in an East Asian population to date—highlights three imprinted genomic domains (*L3MBTL1*, *PEG3*/*ZIM2*/*MIMT1*, *BLCAP*/*NNAT*) as the top-ranked regional methylation signals, of which *L3MBTL1* and *PEG3* are robust to one-carbon-biomarker and genotype adjustment. The findings generate a biologically plausible hypothesis linking one-carbon metabolism dysregulation to altered methylation at imprinting-associated domains. Replication in independent East Asian cohorts is warranted.

**Supplementary Information:**

The online version contains supplementary material available at https://doi.org/10.1186/s13148-026-02227-5.

## Introduction

Elevated plasma homocysteine is a well-established risk factor for cardiovascular disease, stroke, and cognitive decline [1], and is common in middle-aged and older adults worldwide [2]. Despite its clear epidemiological significance, the molecular mechanisms through which elevated homocysteine contributes to organ-level disease remain incompletely understood. Identifying these pathways is central to understanding why hyperhomocysteinemia is harmful and what biological processes it disrupts.

A mechanistically compelling hypothesis is that homocysteine exerts its effects through epigenetic regulation. Homocysteine is a key intermediate in one-carbon metabolism and its accumulation raises intracellular S-adenosylhomocysteine (SAH), a potent inhibitor of DNA methyltransferases [3, 4]. Experimental and observational evidence shows that elevated homocysteine is associated with global DNA hypomethylation [5, 6], raising the possibility that chronic hyperhomocysteinemia alters the methylation—and thereby the regulation—of specific genes throughout the genome. The functional consequence of methylation, however, depends on genomic context: methylation of promoter CpG islands is generally associated with transcriptional silencing, whereas gene-body methylation often correlates positively with expression, and methylation at imprinting control regions and CpG-island shores governs parent-of-origin allele-specific expression. Region-resolved analyses are therefore informative beyond global measures.

Epigenome-wide association studies (EWAS) offer a systematic approach to this question. Prior EWAS have demonstrated that homocysteine-associated methylation differences exist at specific loci in human blood, and Mendelian randomization has provided supporting evidence for a causal direction of effect [7, 8]. However, these studies have been conducted predominantly in European cohorts, and it remains poorly understood whether the same loci—or different ones—are implicated across human populations with varying genetic backgrounds and dietary environments [9]. Genome-wide methylation analyses of homocysteine in East Asian populations remain limited; to our knowledge the principal prior report was conducted in Chinese patients with hypertension using serum homocysteine [10], in contrast to the present community-based population cohort.

In this study, we used genome-wide DNA methylation data from 1,510 adults in the Korea Genome and Epidemiology Study (KoGES/KARE) to conduct an EWAS of fasting plasma homocysteine. We aimed to identify both single-CpG and regional methylation correlates of homocysteine levels, and to compare the resulting signal architecture with prior European EWAS, in order to assess consistency and identify potentially East Asian–specific associations.

## Methods

### Study population

The Korea Genome and Epidemiology Study (KoGES) is a government-funded, multi-site prospective cohort study of the Korean adult population initiated in 2001 [11]. The Korean Association Resource (KARE) sub-cohort recruited community-dwelling adults aged 40–69 years from two communities (Anseong and Ansan) and has followed participants across nine biennial examination waves. From the full KARE cohort (*n* = 10,030), a sub-sample of 1,528 participants with available genome-wide DNA methylation data (Illumina EPIC 850 K array) and fasting plasma homocysteine measurements was included. This sub-sample was selected on the basis of availability of stored peripheral-blood genomic DNA of sufficient quality and quantity for EPIC array profiling under the KARE biospecimen program; it was not a nested case-control selection on any phenotype. To assess potential selection bias, we compared key baseline characteristics of the methylation sub-cohort with the remaining KARE participants (Supplementary Table S3). All participants provided written informed consent. The KoGES study was approved by the Institutional Review Board of the Korea National Institute of Health (KNIH). This secondary analysis was approved by the Institutional Review Board of Incheon St. Mary’s Hospital, The Catholic University of Korea (IRB No. OC25ZASI0053).

### Plasma homocysteine measurement

Fasting plasma total homocysteine was measured by an enzymatic assay at a single central laboratory (Seoul Clinical Laboratories); all participants’ values were determined by the same method, ensuring methodological consistency across the study population. Hyperhomocysteinemia was defined as a fasting plasma concentration > 16 µmol/L [12]. For statistical analysis, homocysteine concentrations were natural log-transformed (LOG_HOMOCYST) to approximate normality and reduce undue leverage from extreme values; the distribution of plasma homocysteine on the raw and log scales is shown in Fig. 3. Seventeen participants with homocysteine concentrations exceeding four standard deviations above the cohort mean (> 37.2 µmol/L, calculated before exclusion) were excluded as extreme outliers, as values at this level may reflect acute illness, rare metabolic disorders, or assay error; one additional participant with missing covariate data was excluded, leaving 1,510 samples for the primary analysis. Homocysteine was measured at a single examination wave; the extent to which a single fasting measurement represents longer-term exposure is uncertain. Covariates included age (years), sex (male = 1), body mass index (BMI, kg/m^2^), and smoking status (never, former, current). Hyperhomocysteinemia status was used only for descriptive classification; all association analyses modelled homocysteine continuously as LOG_HOMOCYST.

### DNA methylation profiling and quality control

Genomic DNA was extracted from peripheral blood leukocytes and methylation was profiled using the Illumina Infinium MethylationEPIC BeadChip (850 K array) [13] according to the manufacturer’s protocol. Raw intensity data (IDAT files) were processed in R using the *minfi* package (version 1.46) [14] with *preprocessNoob* normalization [15] in batches of 50 samples. Processed beta values were stored in a disk-backed HDF5 matrix (HDF5Array package; Bioconductor). Probes failing detection in more than 5% of samples (detection *p* > 0.01) and probes on sex chromosomes were removed, leaving 845,023 CpG sites for analysis. Beta values were clipped to [0.001, 0.999] before logit transformation to M-values (M = log_2_[β/(1–β)]). Standard probe-level filters for SNP-affected probes and published cross-reactive probes [16] were not applied to the primary analysis; however, all FDR-significant probe-level hits and DMR-level top signals were manually verified against these lists, and none were flagged. The array position variable (Sentrix_Position) was included as a covariate to capture the most prominent spatial gradient in signal intensity; chip- or plate-level identifiers were not separately available.

### Blood cell type deconvolution

Blood cell type proportions were estimated using the *EpiDISH* package (version 2.14) [17] with the cent12CT.m 12-cell-type reference matrix and the Robust Partial Correlations (RPC) method. All 1,528 samples yielded complete deconvolution estimates. The neutrophil proportion was excluded from regression to avoid perfect collinearity, leaving 11 cell type proportions as covariates.

### Epigenome-wide association study

At each of 845,023 CpG sites, the M-value was regressed on LOG_HOMOCYST using ordinary least squares, adjusting for age, sex, BMI, smoking status (current and former indicators), array row position (Sentrix_Position), global mean M-value per sample, and 11 blood cell type proportions. The global mean M-value was computed across 10,000 evenly spaced probes (excluding the probe under test) as a sample-level summary of genome-wide methylation load. Because homocysteine may itself influence global methylation levels [4, 5], this covariate may partially attenuate diffuse biological effects; the present results therefore emphasise locus-specific associations. The β coefficient for the homocysteine term and its two-tailed p-value were extracted at each probe. Multiple testing correction used the Benjamini-Hochberg false discovery rate (FDR); epigenome-wide significance was defined as *p* < 1 × 10^−7^ [18]. Genomic inflation was assessed by the inflation factor λ.

### Differentially methylated region analysis

Candidate DMRs were identified by grouping probes within 1,000 bp of one another on the same chromosome—a bandwidth consistent with established EPIC array DMR methods [19]—and requiring at least two probes per region. For each candidate region, probe-level association statistics were combined using Stouffer’s method (Z_i = sign(t) × Φ^−1^(1 − p/2); Z = ΣZ_i/√k). Benjamini-Hochberg FDR correction was applied across all candidate regions. This approach does not explicitly model spatial correlation between adjacent probes, which is a recognised limitation of positional clustering methods.

### Sensitivity analyses

To evaluate the robustness of the top findings to additional determinants of homocysteine and DNA methylation, we re-fit the models with the following covariates added, individually and jointly: (i) alcohol consumption (never/former/current); (ii) serum creatinine (measured by the Jaffe kinetic method, alkaline picrate, on a Hitachi 7600 autoanalyzer); (iii) plasma folate and vitamin B12 (log-transformed; available for *n* = 1,246); and (iv) *MTHFR* C677T genotype. *MTHFR* C677T (rs1801133) was extracted from KARE Affymetrix 5.0 genotype data (proxy SNP_A-2116773; chr1:11,857,429; T-allele frequency 0.49) and coded as the additive count of the 677T allele. The folate/B12-adjusted and *MTHFR*-adjusted analyses were performed genome-wide with the full DMR pipeline and region-level FDR; the alcohol- and creatinine-adjusted analyses were performed on the constituent CpGs of the top DMRs and lead probes. We note that folate and vitamin B12 are upstream determinants of homocysteine within one-carbon metabolism, so their adjustment may partly represent over-adjustment of a mediator; these are therefore reported as sensitivity rather than primary models.

### Annotation and statistical software

CpG sites were annotated using the *IlluminaHumanMethylationEPICanno.ilm10b4.hg19* Bioconductor package (genome build hg19). All analyses were performed in R (version 4.3) using Bioconductor packages including *minfi*, *EpiDISH*, *HDF5Array*, *rhdf5*, and *IlluminaHumanMethylationEPICanno.ilm10b4.hg19*. *MTHFR* genotype extraction used PLINK 2.0. Figures were generated using *ggplot2* (v3.4). Analysis scripts are available from the corresponding author upon reasonable request.

## Results

### Participant characteristics

Of 1,528 eligible KARE participants, 17 (1.1%) were excluded as extreme homocysteine outliers and one for missing covariate data, leaving 1,510 participants for the primary EWAS. Characteristics are summarized in Table 1. Mean age was 59.8 ± 8.8 years; 52.8% were male. Mean BMI was 24.3 ± 3.0 kg/m^2^. Fasting plasma homocysteine was right-skewed (mean 14.19 ± 5.51 µmol/L, median 12.98 [IQR 10.99–15.80] µmol/L; Fig. 3); 22.7% of participants met the criterion for hyperhomocysteinemia (> 16 µmol/L).

**Table 1:** Baseline characteristics of the study population (*n* = 1,510)

Characteristic	Value
*Demographics*	
Age, years	59.8 ± 8.8
Male sex, n (%)	799 (52.8%)
*Anthropometric*	
BMI, kg/m^2^	24.3 ± 3.0
*Lifestyle*	
* Smoking status*	
Never, n (%)	891 (59.0%)
Former, n (%)	340 (22.5%)
Current, n (%)	281 (18.6%)
* Alcohol consumption*	
Never, n (%)	743 (49.2%)
Former, n (%)	79 (5.2%)
Current, n (%)	688 (45.6%)
*Biochemical*	
Plasma homocysteine, µmol/L	14.19 ± 5.51
Median homocysteine [IQR], µmol/L	12.98 [10.99–15.80]
Hyperhomocysteinemia (> 16 µmol/L), n (%)	343 (22.7%)
Serum creatinine, mg/dL	0.95 ± 0.22
Total cholesterol, mg/dL	190.1 ± 34.3
HDL cholesterol, mg/dL	42.9 ± 10.9

Data are mean ± SD or n (%). BMI, body mass index; HDL, high-density lipoprotein; IQR, interquartile range. Hyperhomocysteinemia defined as fasting plasma homocysteine > 16 µmol/L [3]. Homocysteine was measured by an enzymatic assay at a single central laboratory (Seoul Clinical Laboratories). Seventeen participants with homocysteine > 37.2 µmol/L (> 4 SD above cohort mean) were excluded prior to analysis

### Representativeness of the methylation sub-cohort

Compared with the remaining KARE participants (*n* = 8,519), the methylation sub-cohort was closely representative for the exposure of interest and key demographics: mean homocysteine (13.85 vs. 13.92 µmol/L, *p* = 0.61), age (59.7 vs. 60.0 years, *p* = 0.34), and BMI (24.4 vs. 24.5 kg/m^2^, *p* = 0.11) did not differ materially. The sub-cohort included modestly more men (52.4% vs. 45.4%) and current smokers (18.5% vs. 14.3%); because sex and smoking are adjusted covariates in all models, these differences are unlikely to bias the reported associations (Supplementary Table S3).

### Probe-level EWAS results

**Fig. 1:**
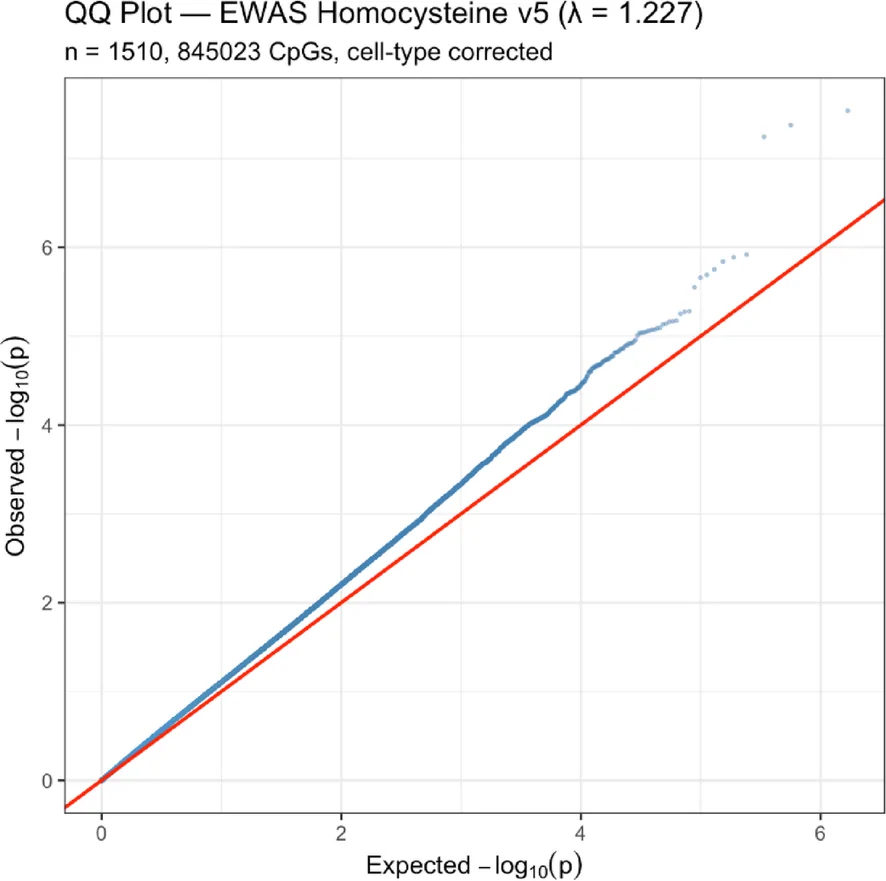
Quantile–quantile (QQ) plot of EWAS p-values. Observed −log_10_(p) versus expected under the null. The genomic inflation factor λ = 1.227. The 95% confidence band is shown in grey

**Fig. 2:**
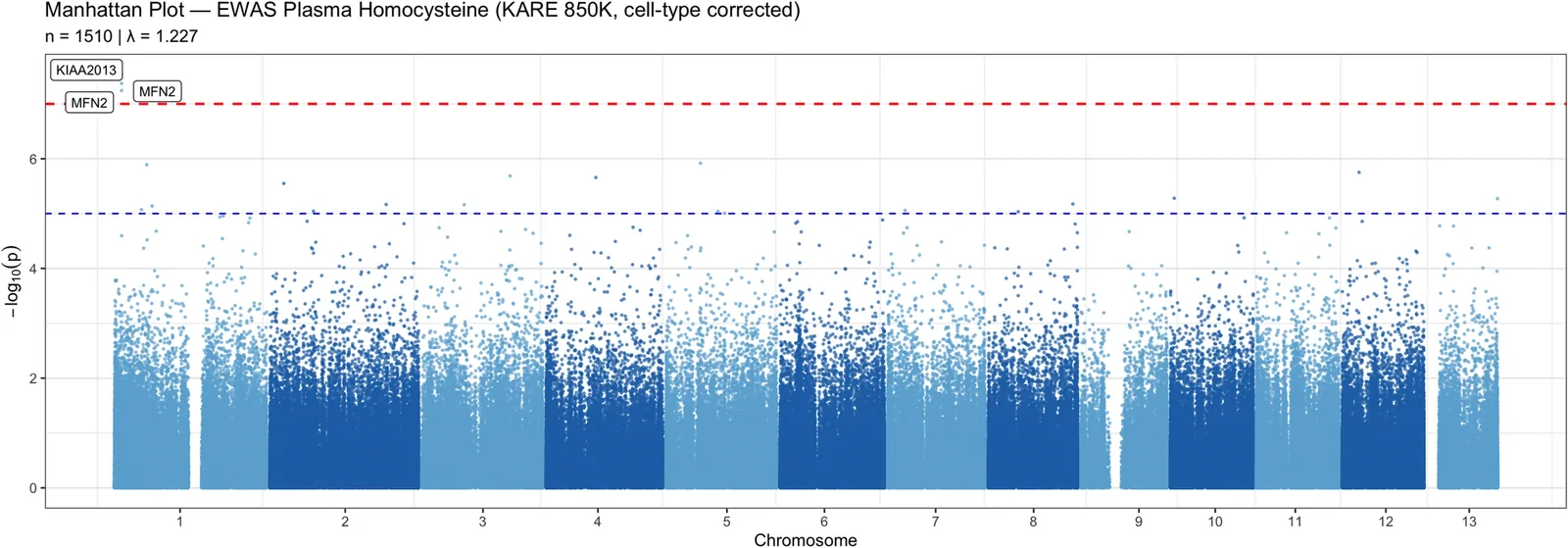
Manhattan plot of EWAS results. −log_10_(p) by chromosomal position for 845,023 CpG sites. Solid line: epigenome-wide significance (*p* = 1 × 10^−7^); dashed line: suggestive threshold (*p* = 1 × 10^−5^). Three probes on chromosome 1p36 exceed the significance threshold

**Fig. 3:**
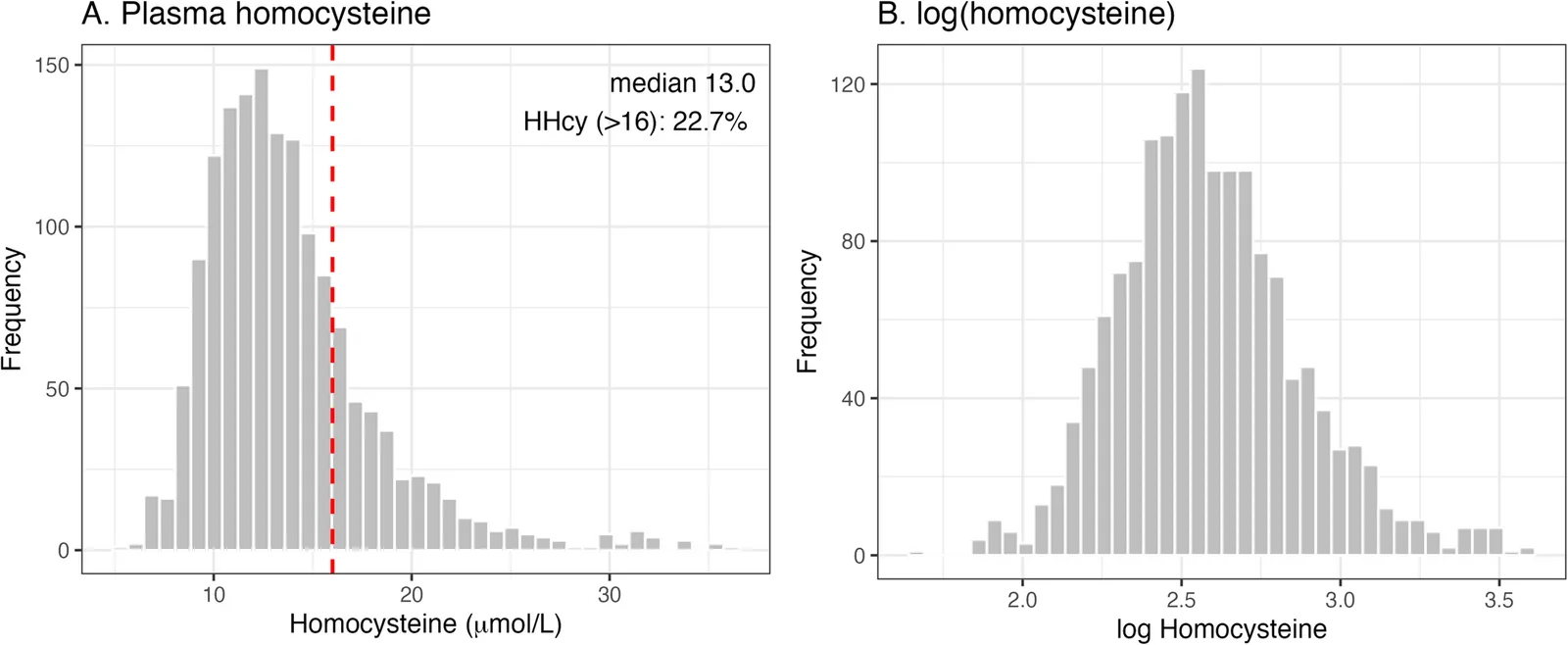
Distribution of plasma homocysteine.** A** Raw plasma homocysteine (µmol/L); the dashed line marks the hyperhomocysteinemia threshold (> 16 µmol/L).** B** Natural-log–transformed homocysteine, approximately normal (skewness 0.49), supporting use of log-homocysteine in regression

After quality filtering, 845,023 CpG sites were tested. The genomic inflation factor was λ = 1.227 (Fig. 1, QQ plot), indicating mild residual inflation consistent with EWAS of a metabolic phenotype with complex biological structure. Three probes reached FDR < 0.05, all on chromosome 1p36 within a ~ 57 kb window: cg07603449 in *KIAA2013* (chr1:11,986,842, β = + 0.140, *p* = 2.91 × 10^−8^, FDR = 0.016), cg10283346 in *MFN2* (chr1:12,044,158, β = − 0.178, *p* = 4.22 × 10^−8^, FDR = 0.016), and cg15199735 in *MFN2* (chr1:12,043,655, β = − 0.186, *p* = 5.70 × 10^−8^, FDR = 0.016). All three exceeded the conventional epigenome-wide threshold of *p* < 1 × 10^−7^. A total of 29 probes surpassed the suggestive threshold of *p* < 1 × 10^−5^ (Supplementary Table S1) (Figs. 2 and 3).

### Regional analysis: differentially methylated regions

The top 15 DMRs are shown in Table 2; all had Benjamini-Hochberg FDR < 0.001. A notable biological pattern emerged: three of the highest-ranked DMRs all map to well-characterised imprinted genomic domains.

**Table 2:** Top 15 differentially methylated regions associated with plasma homocysteine (*n* = 1,510)

Rank	Region	Gene	CpGs, *n*	Width, bp	Direction	Stouffer *p*	Imprinted
1	chr20:42,142,005–42,144,597	*L3MBTL1*	31	2,592	↓	2.0 × 10^−20^	Yes
2	chr19:57,346,735–57,353,128	*PEG3*/*ZIM2*/*MIMT1*	40	6,393	↑	5.2 × 10^−20^	Yes
3	chr20:36,145,902–36,151,683	*BLCAP*/*NNAT*	55	5,781	↓	1.4 × 10^−15^	Yes
4	chr6:29,520,527–29,521,803	HLA region	35	1,276	↑	1.4 × 10^−15^	No
5	chr14:22,942,833–22,948,498	—	17	5,665	↑	5.9 × 10^−15^	No
6	chr5:96,037,871–96,039,870	*CAST*	15	1,999	↑	8.9 × 10^−14^	No
7	chr5:135,414,858–135,416,613	*MIR886*	18	1,755	↓	6.4 × 10^−13^	No
8	chr6:28,910,953–28,912,166	*LINC01556*	14	1,213	↓	6.7 × 10^−12^	No
9	chr11:129,817,347–129,818,685	*PRDM10*	10	1,338	↑	1.3 × 10^−11^	No
10	chr2:43,903,227–43,904,011	*PLEKHH2*	11	784	↑	2.0 × 10^−11^	No
11	chr2:47,382,287–47,383,720	*C2orf61*	14	1,433	↓	2.2 × 10^−11^	No
12	chr19:55,972,504–55,974,513	*ISOC2*	15	2,009	↑	2.7 × 10^−11^	No
13	chr14:22,960,834–22,964,042	—	14	3,208	↑	3.3 × 10^−11^	No
14	chr7:19,812,327–19,813,525	*TMEM196*	18	1,198	↑	3.8 × 10^−11^	No
15	chr15:26,107,382–26,111,090	*ATP10A*	37	3,708	↓	7.9 × 10^−11^	No

Direction: ↑ higher methylation with higher homocysteine; ↓ lower. —, intergenic. All 15 regions had Benjamini-Hochberg FDR < 0.001. The complete DMR list is in Supplementary Table S2

The top-ranked DMR mapped to the L3MBTL1 locus on chromosome 20q12 (chr20:42,142,005–42,144,597, 31 CpGs, width 2,592 bp, mean β = − 0.036, Stouffer *p* = 2.0 × 10^−20^). *L3MBTL1* is a Polycomb group gene that is maternally imprinted (paternally expressed), whose methylation maintenance depends on DNMT3A activity.

The second-ranked DMR encompassed the PEG3 imprinted domain on chromosome 19q13.43 (chr19:57,346,735–57,353,128, 40 CpGs, width 6,393 bp, mean β = + 0.028, Stouffer *p* = 5.2 × 10^−20^), spanning *MIMT1*, *PEG3*, and *ZIM2*, which share a common imprinting control region.

The third-ranked DMR was the BLCAP/NNAT imprinted locus on chromosome 20q11.21 (chr20:36,145,902–36,151,683, 55 CpGs, width 5,781 bp, mean β = − 0.034, Stouffer *p* = 1.4 × 10^−15^). *NNAT* (neuronatin) is paternally expressed and maternally methylated, playing a role in energy homeostasis and pancreatic β-cell function [20].

Additional significant DMRs included *CAST* (chr5), *MIR886* (chr5), *LINC01556* (chr6), *PRDM10* (chr11), *C2orf61* (chr2), *ISOC2* (chr19), and *ATP10A* (chr15; Table 2).

### Sensitivity analyses

The three imprinted DMRs and the three lead probes were re-evaluated under additional adjustments (Supplementary Table S4). The L3MBTL1 and PEG3 domains remained highly significant under every adjustment, including alcohol, serum creatinine, folate/vitamin B12, and *MTHFR* C677T genotype. In the genome-wide *MTHFR*-adjusted analysis (*n* = 1,506, λ = 1.21), *PEG3*/*ZIM2*/*MIMT1* (Stouffer *p* = 4.4 × 10^−20^, FDR = 5.1 × 10^−15^), *L3MBTL1* (*p* = 8.3 × 10^−19^, FDR = 4.9 × 10^−14^), and *BLCAP*/*NNAT* (*p* = 6.9 × 10^−15^, FDR = 2.7 × 10^−10^) all retained strong significance, indicating they are not explained by *MTHFR* genotype. The BLCAP/NNAT domain was markedly attenuated by folate/vitamin B12 adjustment (Stouffer *p* = 5.4 × 10^−17^ → 5.3 × 10^−4^), indicating that this signal is substantially dependent on one-carbon vitamin status.

In contrast, the three chromosome-1 probes (*KIAA2013*, *MFN2*) lost epigenome-wide significance after MTHFR C677T adjustment (FDR 0.016 → 0.38–0.43; no probe reached FDR < 0.05 genome-wide), despite essentially unchanged sample size. These probes lie ~ 130–190 kb from the *MTHFR* C677T variant (chr1:11,856,378), and this pattern is consistent with *cis*-meQTL confounding by the *MTHFR* haplotype—a well-established genetic determinant of plasma homocysteine—rather than a direct metabolic effect of homocysteine.

### Direction of effect

The three imprinted DMRs showed a mixed direction pattern: *L3MBTL1* (mean β = − 0.036) and *BLCAP*/*NNAT* (mean β = − 0.034) were hypomethylated with higher homocysteine, whereas the *PEG3* domain (mean β = + 0.028) was hypermethylated. This bidirectional pattern is consistent with locus-specific perturbation of allele-asymmetric methylation maintenance.

## Discussion

We conducted an epigenome-wide association study of plasma homocysteine in a Korean population—one of the largest population-based homocysteine EWAS reported in an East Asian population to date—analysing 845,023 CpG sites in 1,510 community-dwelling adults from the KoGES/KARE cohort. Three probes on chromosome 1 reached FDR < 0.05, and regional analysis identified three imprinted genomic domains among the top-ranked DMRs: *L3MBTL1* (chr20q12), *PEG3*/*ZIM2*/*MIMT1* (chr19q13.43), and *BLCAP*/*NNAT* (chr20q11.21). Sensitivity analyses showed that the *L3MBTL1* and *PEG3* domains are robust to adjustment for alcohol, renal function, folate, vitamin B12, and *MTHFR* genotype, whereas the chromosome-1 probe-level signals are most likely attributable to *cis*-genetic confounding. These findings extend the homocysteine methylation map to an East Asian population and generate a biologically plausible hypothesis linking one-carbon metabolism dysregulation to altered methylation at imprinting-associated domains, warranting further mechanistic investigation. We do not claim that these loci are intrinsically more dependent on DNMT activity than other genomic regions; establishing such preferential dependence would require direct functional and allele-specific data.

The recurrence of three known imprinted domains among the highest-ranked DMRs is biologically intriguing. Imprinted DMRs are established and maintained by DNMT3A, DNMT1, and DNMT3L through a continuous process requiring SAM as the methyl donor [21, 22], and SAH—which accumulates when homocysteine is elevated—competitively inhibits these enzymes. Because imprinted DMRs require strict allele-asymmetric methylation, even modest perturbation of the SAM/SAH ratio may produce detectable shifts at these loci. However, this interpretation rests on bulk blood methylation data, and the observed associations could alternatively reflect locus-specific chromatin properties, co-regulation by nearby elements, or residual cell-type compositional differences.

That the *L3MBTL1* and *PEG3* domains remained significant after adjustment for folate, vitamin B12, and *MTHFR* C677T genotype argues against their being driven by vitamin status or this major genetic determinant of homocysteine, and supports their specificity. To our knowledge, *L3MBTL1* has not been previously reported in homocysteine EWAS. The *BLCAP*/*NNAT* signal, by contrast, was substantially attenuated by folate/B12 adjustment, suggesting that its association with homocysteine operates largely through the folate/B12–homocysteine axis of one-carbon metabolism.

A key new observation concerns the probe-level chromosome-1 signals. The three FDR-significant probes (*KIAA2013*, *MFN2*) lie ~ 130–190 kb from the *MTHFR* C677T variant and lost epigenome-wide significance after C677T adjustment, with no genome-wide probe reaching FDR < 0.05. This pattern is most parsimoniously explained by *cis*-meQTL confounding: *MTHFR* C677T influences both plasma homocysteine and local methylation, generating a non-causal probe–homocysteine association. The imprinted DMRs, which lie far from *MTHFR* and were unaffected by C677T adjustment, are thereby distinguished from this genetic confounding.

The top hits from the largest published European homocysteine EWAS (Dekkers et al., *n* = 2,035 [7])—*SLC27A1*, *AJUBA*, *KCNMA1*—were not replicated at FDR < 0.05. Non-replication may reflect population differences in epigenetic architecture, allele frequencies, dietary environment, and cohort characteristics; notably, the KARE cohort comprises Korean adults among whom elevated homocysteine is common [23]. The EPIC 850 K array provides denser coverage of imprinted regions than the 450 K array used in Dekkers et al., which may partly explain detection of imprinted-domain DMR signals.

The genomic inflation factor λ = 1.227 likely reflects genuine polygenicity, residual cell-type confounding despite 11-cell-type EpiDISH adjustment, and unmeasured dietary confounders. This level of inflation is commonly observed in well-conducted EWAS of metabolic traits [18]. Several limitations warrant consideration. First, the cross-sectional design precludes causal inference, and homocysteine was measured at a single examination wave. Second, DNA methylation was measured in peripheral blood and does not directly reflect target tissues. Third, the regional signals are based on bulk blood methylation, and allele-specific effects cannot be determined from array data alone. Fourth, homocysteine was measured by a standardized enzymatic assay rather than the HPLC with electrochemical detection reference method, although a single central laboratory ensured methodological consistency. Finally, the DMR analysis does not model spatial correlation between adjacent CpGs.

In conclusion, this EWAS of plasma homocysteine in a Korean cohort identifies three imprinted genomic domains among the strongest regional methylation signals—*L3MBTL1*, *PEG3*/*ZIM2*/*MIMT1*, and *BLCAP*/*NNAT*—of which *L3MBTL1* and *PEG3* are robust to one-carbon-biomarker and genotype adjustment, while the probe-level chromosome-1 signals most likely reflect *cis*-meQTL confounding by *MTHFR*. We propose, as a hypothesis for future testing, that the SAM/SAH ratio rather than homocysteine concentration per se may be the more proximal regulator of methylation maintenance at the affected loci; direct measurement of SAM and SAH, allele-specific methylation studies, and replication in independent East Asian cohorts will be required to test this.

## Supplementary Information

Below is the link to the electronic supplementary material.

Supplementary Material 1

## Data Availability

The DNA methylation and phenotypic data used in this study were obtained from the Korea Genome and Epidemiology Study (KoGES/KARE) cohort, which is managed by the Korea National Institute of Health (KNIH). These data are not publicly available due to participant privacy protections and data governance policies. Qualified researchers may apply for access through the Korean Genome and Epidemiology Study data repository (https://www.koreabiobank.re.kr). The analysis code used in this study is available from the corresponding author upon reasonable request.
