# Supplementary material for: Epigenome-wide association study of plasma homocysteine in a Korean cohort highlights imprinted genomic domains as top regional methylation signals
Source: Clin Epigenetics. 2026 Aug 12;18:208. doi: 10.1186/s13148-026-02227-5 (PMC13749688; doi:10.1186/s13148-026-02227-5)
Supplement: Supplementary file 1 [file 13148_2026_2227_MOESM1_ESM.docx]

# Supplementary Material

*Epigenome-wide association study of plasma homocysteine in a Korean cohort highlights imprinted genomic domains as top regional methylation signals*

Jaeeun Yoo, MD, PhD

## Supplementary Table S1. Probe-level associations surpassing the suggestive threshold (*P* < 1 × 10⁻⁵) in the primary EWAS

Single-CpG associations with log-transformed plasma homocysteine (n = 1,510), adjusted for age, sex, BMI, smoking, array position, global mean methylation, and 11 blood cell-type proportions. β, M-value regression coefficient per unit log-homocysteine; ↑/↓, higher/lower methylation with higher homocysteine; FDR, Benjamini–Hochberg. Probes 1–3 reach epigenome-wide significance (FDR < 0.05). The complete probe-level summary statistics for all 845,023 CpGs are deposited at Zenodo (DOI 10.5281/zenodo.19642241).

| # | CpG | Chr:position | Gene | Island | β | *P* | FDR |
| --- | --- | --- | --- | --- | --- | --- | --- |
| 1 | cg07603449 | chr1:11,986,842 | *KIAA2013* | S_Shore | +0.140 ↑ | 2.91 × 10⁻⁸ | 0.016 |
| 2 | cg10283346 | chr1:12,044,158 | *MFN2* | S_Shelf | −0.178 ↓ | 4.22 × 10⁻⁸ | 0.016 |
| 3 | cg15199735 | chr1:12,043,655 | *MFN2* | S_Shelf | −0.186 ↓ | 5.70 × 10⁻⁸ | 0.016 |
| 4 | cg06740703 | chr5:56,152,491 | *MAP3K1* | OpenSea | +0.098 ↑ | 1.21 × 10⁻⁶ | 0.203 |
| 5 | cg01381805 | chr1:53,040,388 | *—* | OpenSea | +0.102 ↑ | 1.29 × 10⁻⁶ | 0.203 |
| 6 | cg06871497 | chr19:2,087,708 | *MOB3A* | OpenSea | −0.172 ↓ | 1.44 × 10⁻⁶ | 0.203 |
| 7 | cg20565652 | chr12:27,687,275 | *PPFIBP1* | OpenSea | +0.054 ↑ | 1.77 × 10⁻⁶ | 0.206 |
| 8 | cg11887851 | chr3:144,304,512 | *—* | OpenSea | +0.133 ↑ | 2.05 × 10⁻⁶ | 0.206 |
| 9 | cg19143170 | chr4:81,303,914 | *C4orf22* | N_Shelf | +0.155 ↑ | 2.20 × 10⁻⁶ | 0.206 |
| 10 | cg26445167 | chr2:22,754,600 | *—* | OpenSea | +0.114 ↑ | 2.82 × 10⁻⁶ | 0.239 |
| 11 | cg07091793 | chr10:6,362,483 | *—* | OpenSea | +0.106 ↑ | 5.25 × 10⁻⁶ | 0.279 |
| 12 | cg12673774 | chr13:114,960,970 | *—* | N_Shelf | +0.109 ↑ | 5.34 × 10⁻⁶ | 0.279 |
| 13 | cg18247396 | chr21:33,092,959 | *SCAF4* | OpenSea | +0.099 ↑ | 5.60 × 10⁻⁶ | 0.279 |
| 14 | cg04810707 | chr8:138,161,113 | *—* | OpenSea | +0.119 ↑ | 6.70 × 10⁻⁶ | 0.279 |
| 15 | cg23165166 | chr2:189,914,501 | *COL5A2* | OpenSea | +0.127 ↑ | 6.81 × 10⁻⁶ | 0.279 |
| 16 | cg18780425 | chr3:69,250,024 | *FRMD4B* | OpenSea | +0.124 ↑ | 6.88 × 10⁻⁶ | 0.279 |
| 17 | cg07417874 | chr1:61,946,752 | *—* | OpenSea | +0.059 ↑ | 7.28 × 10⁻⁶ | 0.279 |
| 18 | cg05266687 | chr19:50,879,559 | *NR1H2* | N_Shore | −0.138 ↓ | 7.41 × 10⁻⁶ | 0.279 |
| 19 | cg20368904 | chr14:103,593,088 | *TNFAIP2* | N_Shore | −0.164 ↓ | 8.09 × 10⁻⁶ | 0.279 |
| 20 | cg15957688 | chr14:22,956,176 | *—* | OpenSea | +0.118 ↑ | 8.19 × 10⁻⁶ | 0.279 |
| 21 | cg15657287 | chr1:44,741,964 | *ERI3* | OpenSea | +0.101 ↑ | 8.47 × 10⁻⁶ | 0.279 |
| 22 | cg24183514 | chr14:51,863,868 | *—* | OpenSea | +0.072 ↑ | 8.48 × 10⁻⁶ | 0.279 |
| 23 | cg07728579 | chr15:83,475,013 | *FSD2* | N_Shelf | +0.115 ↑ | 8.64 × 10⁻⁶ | 0.279 |
| 24 | cg06624056 | chr7:28,319,499 | *LOC402644* | OpenSea | +0.133 ↑ | 8.75 × 10⁻⁶ | 0.279 |
| 25 | cg09938016 | chr2:71,158,623 | *VAX2* | OpenSea | +0.104 ↑ | 9.04 × 10⁻⁶ | 0.279 |
| 26 | cg15253642 | chr5:84,741,535 | *—* | OpenSea | +0.133 ↑ | 9.10 × 10⁻⁶ | 0.279 |
| 27 | cg11920943 | chr8:49,037,460 | *—* | OpenSea | +0.110 ↑ | 9.21 × 10⁻⁶ | 0.279 |
| 28 | cg12909186 | chr15:81,603,948 | *IL16* | OpenSea | +0.133 ↑ | 9.25 × 10⁻⁶ | 0.279 |
| 29 | cg20753349 | chr5:95,583,169 | *LOC101929710* | OpenSea | +0.101 ↑ | 9.84 × 10⁻⁶ | 0.285 |

## Supplementary Table S2. Top 50 differentially methylated regions associated with plasma homocysteine

Regions identified by positional clustering (≤ 1,000 bp gap, ≥ 2 CpGs) with Stouffer-combined p-values and Benjamini–Hochberg region-level FDR. Dir, direction (↑/↓ higher/lower methylation with higher homocysteine). The three imprinted domains (ranks 1–3) are described in the main text (Table 2). The complete list of all candidate DMRs (1,120 at FDR < 0.05) is deposited at Zenodo (DOI 10.5281/zenodo.19642241).

| # | Region | Gene(s) | CpGs | Width (bp) | Dir | Stouffer *p* | FDR |
| --- | --- | --- | --- | --- | --- | --- | --- |
| 1 | chr20:42,142,005–42,144,597 | *L3MBTL/L3MBTL1* | 31 | 2,592 | ↓ | 1.99 × 10⁻²⁰ | 2.36 × 10⁻¹⁵ |
| 2 | chr19:57,346,735–57,353,128 | *MIMT1/PEG3/ZIM2* | 40 | 6,393 | ↑ | 5.23 × 10⁻²⁰ | 3.10 × 10⁻¹⁵ |
| 3 | chr20:36,145,902–36,151,683 | *BLCAP/NNAT* | 55 | 5,781 | ↓ | 1.35 × 10⁻¹⁵ | 4.12 × 10⁻¹¹ |
| 4 | chr6:29,520,527–29,521,803 | *—* | 35 | 1,276 | ↑ | 1.39 × 10⁻¹⁵ | 4.12 × 10⁻¹¹ |
| 5 | chr14:22,942,833–22,948,498 | *—* | 17 | 5,665 | ↑ | 5.86 × 10⁻¹⁵ | 1.39 × 10⁻¹⁰ |
| 6 | chr5:96,037,871–96,039,870 | *CAST* | 15 | 1,999 | ↑ | 8.90 × 10⁻¹⁴ | 1.75 × 10⁻⁹ |
| 7 | chr5:135,414,858–135,416,613 | *MIR886* | 18 | 1,755 | ↓ | 6.43 × 10⁻¹³ | 1.09 × 10⁻⁸ |
| 8 | chr6:28,910,953–28,912,166 | *LINC01556* | 14 | 1,213 | ↓ | 6.73 × 10⁻¹² | 9.96 × 10⁻⁸ |
| 9 | chr11:129,817,347–129,818,685 | *PRDM10* | 10 | 1,338 | ↑ | 1.33 × 10⁻¹¹ | 1.75 × 10⁻⁷ |
| 10 | chr2:43,903,227–43,904,011 | *C1GALT1C1L/LOC728819/PLEKHH2* | 11 | 784 | ↑ | 1.97 × 10⁻¹¹ | 2.34 × 10⁻⁷ |
| 11 | chr2:47,382,287–47,383,720 | *C2orf61* | 14 | 1,433 | ↓ | 2.18 × 10⁻¹¹ | 2.34 × 10⁻⁷ |
| 12 | chr19:55,972,504–55,974,513 | *ISOC2* | 15 | 2,009 | ↑ | 2.65 × 10⁻¹¹ | 2.61 × 10⁻⁷ |
| 13 | chr14:22,960,834–22,964,042 | *—* | 14 | 3,208 | ↑ | 3.28 × 10⁻¹¹ | 2.98 × 10⁻⁷ |
| 14 | chr7:19,812,327–19,813,525 | *TMEM196* | 18 | 1,198 | ↑ | 3.84 × 10⁻¹¹ | 3.24 × 10⁻⁷ |
| 15 | chr15:26,107,382–26,111,090 | *ATP10A* | 37 | 3,708 | ↓ | 7.90 × 10⁻¹¹ | 5.95 × 10⁻⁷ |
| 16 | chr1:16,846,251–16,847,839 | *—* | 12 | 1,588 | ↓ | 8.05 × 10⁻¹¹ | 5.95 × 10⁻⁷ |
| 17 | chr7:127,910,494–127,912,372 | *—* | 9 | 1,878 | ↓ | 8.73 × 10⁻¹¹ | 6.07 × 10⁻⁷ |
| 18 | chr10:99,734,081–99,735,202 | *CRTAC1* | 10 | 1,121 | ↓ | 1.32 × 10⁻¹⁰ | 8.58 × 10⁻⁷ |
| 19 | chr12:47,219,626–47,221,046 | *SLC38A4* | 14 | 1,420 | ↓ | 1.38 × 10⁻¹⁰ | 8.58 × 10⁻⁷ |
| 20 | chr16:85,518,792–85,521,437 | *—* | 14 | 2,645 | ↓ | 1.53 × 10⁻¹⁰ | 9.04 × 10⁻⁷ |
| 21 | chr14:37,123,678–37,136,391 | *PAX9* | 65 | 12,713 | ↑ | 1.64 × 10⁻¹⁰ | 9.25 × 10⁻⁷ |
| 22 | chr13:36,870,809–36,873,065 | *C13orf38/CCDC169/CCDC169-SOHLH2* | 14 | 2,256 | ↓ | 1.72 × 10⁻¹⁰ | 9.25 × 10⁻⁷ |
| 23 | chr17:80,401,500–80,404,898 | *C17orf62* | 15 | 3,398 | ↓ | 2.25 × 10⁻¹⁰ | 1.16 × 10⁻⁶ |
| 24 | chr5:178,986,131–178,987,429 | *RUFY1* | 15 | 1,298 | ↓ | 3.63 × 10⁻¹⁰ | 1.79 × 10⁻⁶ |
| 25 | chr20:61,508,481–61,509,768 | *DIDO1* | 5 | 1,287 | ↓ | 4.28 × 10⁻¹⁰ | 2.02 × 10⁻⁶ |
| 26 | chr10:135,202,522–135,203,200 | *PAOX* | 8 | 678 | ↑ | 4.59 × 10⁻¹⁰ | 2.09 × 10⁻⁶ |
| 27 | chr12:4,916,913–4,920,613 | *KCNA6* | 20 | 3,700 | ↑ | 5.63 × 10⁻¹⁰ | 2.39 × 10⁻⁶ |
| 28 | chr11:1,888,965–1,893,975 | *LSP1* | 28 | 5,010 | ↓ | 5.67 × 10⁻¹⁰ | 2.39 × 10⁻⁶ |
| 29 | chr11:65,321,226–65,323,526 | *LTBP3* | 9 | 2,300 | ↓ | 7.30 × 10⁻¹⁰ | 2.98 × 10⁻⁶ |
| 30 | chr5:131,280,217–131,282,885 | *MEIKIN* | 14 | 2,668 | ↓ | 7.93 × 10⁻¹⁰ | 3.12 × 10⁻⁶ |
| 31 | chr5:140,528,785–140,531,865 | *PCDHB6* | 22 | 3,080 | ↑ | 8.66 × 10⁻¹⁰ | 3.24 × 10⁻⁶ |
| 32 | chr6:3,848,218–3,851,517 | *FAM50B* | 37 | 3,299 | ↓ | 8.76 × 10⁻¹⁰ | 3.24 × 10⁻⁶ |
| 33 | chr10:135,049,208–135,052,004 | *VENTX* | 39 | 2,796 | ↑ | 9.71 × 10⁻¹⁰ | 3.48 × 10⁻⁶ |
| 34 | chr12:120,651,746–120,654,588 | *PXN* | 13 | 2,842 | ↓ | 1.60 × 10⁻⁹ | 5.54 × 10⁻⁶ |
| 35 | chr19:14,606,390–14,608,092 | *GIPC1* | 13 | 1,702 | ↑ | 1.64 × 10⁻⁹ | 5.54 × 10⁻⁶ |
| 36 | chr20:57,462,798–57,467,811 | *GNAS* | 69 | 5,013 | ↑ | 1.79 × 10⁻⁹ | 5.71 × 10⁻⁶ |
| 37 | chr5:176,522,511–176,523,370 | *FGFR4* | 6 | 859 | ↓ | 1.79 × 10⁻⁹ | 5.71 × 10⁻⁶ |
| 38 | chr14:22,923,261–22,924,469 | *—* | 6 | 1,208 | ↑ | 2.16 × 10⁻⁹ | 6.73 × 10⁻⁶ |
| 39 | chr11:32,416,490–32,417,943 | *WT1* | 9 | 1,453 | ↑ | 2.78 × 10⁻⁹ | 8.42 × 10⁻⁶ |
| 40 | chr10:43,047,134–43,048,734 | *ZNF37B* | 18 | 1,600 | ↓ | 3.79 × 10⁻⁹ | 1.12 × 10⁻⁵ |
| 41 | chr14:34,268,685–34,270,437 | *NPAS3* | 9 | 1,752 | ↓ | 3.99 × 10⁻⁹ | 1.15 × 10⁻⁵ |
| 42 | chr5:1,083,885–1,094,035 | *SLC12A7* | 48 | 10,150 | ↓ | 4.53 × 10⁻⁹ | 1.28 × 10⁻⁵ |
| 43 | chr1:42,383,598–42,385,941 | *HIVEP3* | 18 | 2,343 | ↓ | 4.66 × 10⁻⁹ | 1.28 × 10⁻⁵ |
| 44 | chr10:134,913,608–134,915,859 | *ADGRA1/GPR123* | 13 | 2,251 | ↓ | 7.31 × 10⁻⁹ | 1.97 × 10⁻⁵ |
| 45 | chr1:1,092,940–1,106,175 | *MIR200A/MIR200B/MIR429* | 46 | 13,235 | ↓ | 7.89 × 10⁻⁹ | 2.07 × 10⁻⁵ |
| 46 | chr18:74,960,629–74,963,807 | *GALR1* | 37 | 3,178 | ↑ | 8.26 × 10⁻⁹ | 2.12 × 10⁻⁵ |
| 47 | chr11:31,824,327–31,841,980 | *DKFZp686K1684/PAX6* | 101 | 17,653 | ↑ | 1.18 × 10⁻⁸ | 2.97 × 10⁻⁵ |
| 48 | chr4:56,023,423–56,024,434 | *—* | 6 | 1,011 | ↑ | 1.43 × 10⁻⁸ | 3.52 × 10⁻⁵ |
| 49 | chr6:30,420,981–30,421,655 | *—* | 17 | 674 | ↑ | 2.36 × 10⁻⁸ | 5.70 × 10⁻⁵ |
| 50 | chr5:56,147,444–56,148,274 | *MAP3K1* | 3 | 830 | ↑ | 2.72 × 10⁻⁸ | 6.31 × 10⁻⁵ |

## Supplementary Table S3. Comparison of the methylation sub-cohort with the remaining KARE cohort

Assessment of potential selection bias and generalizability. Values are mean (SD) for continuous variables or % for categorical variables. p-values from two-sample t-test (continuous) or χ² test (categorical).

| Variable | Methylation sub-cohort (n = 1,511) | Rest of KARE (n = 8,519) | *p* |
| --- | --- | --- | --- |
| Age, years | 59.7 (8.8) | 60.0 (8.6) | 0.34 |
| Male sex, % | 52.4 | 45.4 | 2.1 × 10⁻⁶ |
| BMI, kg/m² | 24.4 (3.0) | 24.5 (3.1) | 0.11 |
| Plasma homocysteine, µmol/L | 13.85 (4.36) | 13.92 (5.31) | 0.61 |
| Serum creatinine, mg/dL | 0.95 (0.21) | 0.93 (0.22) | 0.013 |
| Current smoker, % | 18.5 | 14.3 | 6.9 × 10⁻⁵ |
| Current drinker, % | 45.6 | 43.1 | 0.094 |

The full KARE cohort comprised 10,030 participants. The methylation sub-cohort is closely representative for homocysteine, age, and BMI; it includes modestly more men and current smokers, both of which are adjusted in all association models.

## Supplementary Table S4. Sensitivity analyses: significance of the top imprinted DMRs and lead probes under additional covariate adjustments

**(a) Imprinted DMRs — Stouffer combined p-value by model.**

| DMR | Primary | + Alcohol | + Creatinine | + Folate/B12 | + *MTHFR* C677T | Fully adjusted |
| --- | --- | --- | --- | --- | --- | --- |
| *L3MBTL1* | 1.2 × 10⁻²¹ | 7.1 × 10⁻²² | 5.6 × 10⁻²³ | 8.8 × 10⁻¹⁵ | 4.9 × 10⁻²⁰ | 2.0 × 10⁻¹³ |
| *PEG3*/*ZIM2*/*MIMT1* | 1.0 × 10⁻¹⁸ | 6.4 × 10⁻¹⁹ | 5.1 × 10⁻²¹ | 3.1 × 10⁻²¹ | 9.7 × 10⁻¹⁹ | 3.3 × 10⁻²⁶ |
| *BLCAP*/*NNAT* | 5.4 × 10⁻¹⁷ | 3.7 × 10⁻¹⁶ | 5.2 × 10⁻¹⁹ | 5.3 × 10⁻⁴ | 2.7 × 10⁻¹⁶ | 5.5 × 10⁻⁴ |

Sample sizes: n = 1,510 (primary, alcohol, creatinine); n = 1,246 (folate/B12, fully adjusted); n = 1,506 (*MTHFR*). Values are from re-fitting the constituent CpGs of each DMR; the folate/B12- and *MTHFR*-adjusted results were additionally confirmed genome-wide with region-level FDR (panel c).

**(b) Lead chromosome-1 probes — p-value (FDR) by model.**

| Probe (gene) | Primary | + *MTHFR* C677T (genome-wide) |
| --- | --- | --- |
| cg07603449 (*KIAA2013*) | 2.9 × 10⁻⁸ (0.016) | 1.1 × 10⁻⁴ (0.43) |
| cg10283346 (*MFN2*) | 4.2 × 10⁻⁸ (0.016) | 3.9 × 10⁻⁵ (0.38) |
| cg15199735 (*MFN2*) | 5.7 × 10⁻⁸ (0.016) | 1.1 × 10⁻⁴ (0.43) |

After *MTHFR* C677T adjustment, none of the three probes retained epigenome-wide significance, and no probe genome-wide reached FDR < 0.05. The probes lie ~130–190 kb from the *MTHFR* C677T variant (chr1:11,856,378).

**(c) Genome-wide *MTHFR* C677T–adjusted DMR analysis (n = 1,506, λ = 1.21).**

| DMR | Stouffer p | Region-level FDR |
| --- | --- | --- |
| *PEG3*/*ZIM2*/*MIMT1* | 4.4 × 10⁻²⁰ | 5.1 × 10⁻¹⁵ |
| *L3MBTL1* | 8.3 × 10⁻¹⁹ | 4.9 × 10⁻¹⁴ |
| *BLCAP*/*NNAT* | 6.9 × 10⁻¹⁵ | 2.7 × 10⁻¹⁰ |

*MTHFR* C677T (rs1801133) was extracted from KARE Affymetrix 5.0 genotype data (proxy SNP_A-2116773; T-allele frequency 0.49) and coded as the additive 677T-allele count. Correlations of log-homocysteine with one-carbon biomarkers: folate r = −0.33, vitamin B12 r = −0.25, serum creatinine r = +0.45.
